# Medicine

**Published:** 1900-03

**Authors:** Theodore Fisher


### Progress of the Medical Sciences.

### MEDICINE.

That the **diet in typhoid fever**[1] should be of the lightest possible character is, perhaps, considered one of the most reasonable dogmas of the science of medicine. That this dogma should be fixed and unalterable there seems to be some reason to doubt. An interesting paper has recently appeared by Dr. Morris Manges on the subject. Statistics are given showing the percentage of recoveries on a diet containing solid food, and that complications and relapses are not more common. A paper by Barrs, of Leeds,[2] was one of the first on the subject. Since then papers dealing with larger numbers of cases have appeared, the most important by Bushuyeff, a Russian physician. During the year 1897, 318 cases were allowed a liberal diet. Of these 26 died, or 8.2 per cent. The average of deaths for the preceding ten years had been 12.4 per cent. Shattock has also treated 146 cases on a comparatively full diet, with a mortality of 8.1 per cent.

The following are articles of diet given, in the order allowed, in one day by Bushuyeff: Tea with roll, oatmeal, barley or wheat porridge, one or two boiled eggs, a glass of milk with

[1] *Med. Rec.*, 1900, lvii. [2] *Brit. M. J.*, 1897, i. 125.

roll, one half a cutlet and a bit of boiled meat, a plate of chicken broth, tea with roll, a cup of chicken or beef soup, semolina pudding, a piece of chicken, milk with roll. During the night, coffee or tea, with milk, given two to four times. The condition of patients on the full diet is said to be very much better than that of those on a more liquid diet, but it is not possible to persuade all patients to eat.

There is no evidence that on a liberal diet hemorrhages or perforation are more frequent, nor that relapses are more common.

The grounds on which a full diet is justifiable are afterwards briefly discussed. It is remarked that typhoid fever is a general, not a local, disease, and that bacilli are to be found in the blood as early as the fifth day of the disease. It is considered, also, that there is little pathological foundation for the idea that every effort should be made to secure the utmost possible rest for the intestine. There is no reason to think that peristalsis can do harm to the ulcers. While increased peristalsis as a consequence of the more liberal diet can do no harm, ulceration is far less likely to be extensive in a well-nourished than in an ill-nourished body. It has also to be remembered that ulceration may be very slight or absent altogether, while in some cases even swelling of the Peyer's patches or solitary follicles does not occur.

Experience in the *post-mortem* room might suggest that in one way a full diet would introduce an element of danger. With the increased diet a larger mass of fæces could be held above the ileo-cæcal valve. It is in the ileum immediately above this valve that the ulceration almost invariably takes place. The severity of the ulceration in this neighbourhood is probably due to the fact that the delay of the fæces with the numerous micro-organisms they contain allows the micro-organisms to overcome the resistance of the Peyer's patches more effectually than higher up in the small intestine. If delay of fæces at the ileo-cæcal valve is the cause of severe ulceration, then one would think that the full diet should be accompanied by treatment with aperients.

In connection with the subject of the invasion of the intestinal wall by bacilli, it may be interesting to refer to a recent paper by Adami.[1] He briefly refers to the work of various observers, as well as to his own, showing that the Peyer's patches destroy a great number of the bacilli, but that too large a number may be present to be dealt with. Some then enter the portal circulation, and are attacked by the epithelial cells of the liver. Through the liver, however, some micro-organisms pass into the general circulation, to be dealt with by the cells of the convoluted tubules of the kidneys. These facts are not new, but it is well to bear them in mind when considering the question of the more liberal diet in

[1] *Med. News*, 1900, lxxvi. 8.

typhoid fever. Where cells in many parts of the body are striving to overcome bacilli, it is necessary that they should be well provided with nutrient support.

Dr. Adami does not, however, refer to typhoid fever. His paper has more distinct reference to the **production of hæmochromatosis and anæmia** by the invasion of the body by micro-organisms. In hæmochromatosis associated with cirrhosis of the liver, Adami mentions that the probable sequence of events is the entry of micro-organisms into the portal circulation, with consequent inflammatory changes in the liver, associated with blood-destruction. Later, inflammatory changes occur in other organs, especially the pancreas. When fibrosis of the pancreas becomes advanced, diabetes results. Adami mentions that Dr. Mause E. Abbott has confirmed this view of the causation of hæmochromatosis by showing that the masses of pigment can be seen to contain large numbers of micro-organisms.

Two cases of **hæmochromatosis** [1] associated with cirrhosis of the liver have recently been recorded by Osler. One occurred in a man aged 48, the other in a man aged 34.

A few papers have appeared recently on the subject of **albuminuria in the apparently healthy.** [2] An interesting point mentioned is the occurrence of albumin in the urine after severe exercise. Thus 83 specimens of urine were taken by Dr. Darling from oarsmen at the Harvard University, when in training for races, and examined for albumin. Of these 83 specimens, 48 contained albumin. After time-rows and races, the urine was found to be invariably albuminous. In one instance the amount was as high as 0.9 per cent. Dr. Herbert Hawkins [3] also refers to albuminuria after exertion. He mentions that, at St. Thomas's Hospital, Dr. Jenner examined the urine of nine members of the Rugby football team after playing in the final cup tie. In every instance albumin was present, and in some cases hyaline casts also. A paper by Mr. Levison [4] tends to show that in slight traces albumin is met with extremely commonly in the urine, especially at one period of life—that is, between the ages of 18 and 25 years. The urine was found to be albuminous in 45.5 per cent. of 129 hospital patients between the above-mentioned ages. One is tempted to imagine that the albumin in the majority of these cases must have been present in a very minute trace. Levison does not seem altogether to admit this. He considers that probably in 25 to 30 per cent. of the cases albumin would have been recognised in the urine by ordinary clinical methods. Since these cases were hospital patients, and the results in some degree open to criticism on that ground, specimens of urine from 108 soldiers were examined. Here not only was the frequency of the presence of albumin

[1] *Brit. M. J.*, 1899, ii. 1595. [2] *Boston M. & S. J.*, 1899, cxli. 205.

[3] *Brit. M. J.*, 1899, ii. 1598. [4] *St. Barth. Hosp. Rep.* 1899 xxxv. 169.

again made manifest, but the effect of exercise in producing albuminuria was illustrated. Albumin was present in 41.73 per cent. of specimens of urine taken before drill, and in 63.23 per cent. of cases after it.

THEODORE FISHER.

